# The Utility of a Novel Electrocardiogram Patch Using Dry Electrodes Technology for Arrhythmia Detection During Exercise and Prolonged Monitoring: Proof-of-Concept Study

**DOI:** 10.2196/49346

**Published:** 2023-11-30

**Authors:** Lonneke A Fruytier, Daan M Janssen, Israel Campero Jurado, Danny AJP van de Sande, Ilde Lorato, Shavini Stuart, Pradeep Panditha, Margreet de Kok, Hareld MC Kemps

**Affiliations:** 1 Department of Cardiology Máxima MC Eindhoven/Veldhoven Veldhoven Netherlands; 2 Department of Mathematics and Computer Science Eindhoven University of Technology Eindhoven Netherlands; 3 Department of Cardiology University Medical Center Utrecht Utrecht Netherlands; 4 Stichting imec Nederland Eindhoven Netherlands; 5 TNO Holst Centre Eindhoven Netherlands; 6 Department of Industrial Design Eindhoven University of Technology Eindhoven Netherlands

**Keywords:** arrhythmia detection, coronary artery disease, ECG monitoring, electrocardiogram, exercise, patch, usability

## Abstract

**Background:**

Accurate detection of myocardial ischemia and arrhythmias during free-living exercise could play a pivotal role in screening and monitoring for the prevention of exercise-related cardiovascular events in high-risk populations. Although remote electrocardiogram (ECG) solutions are emerging rapidly, existing technology is neither designed nor validated for continuous use during vigorous exercise.

**Objective:**

In this proof-of-concept study, we evaluated the usability, signal quality, and accuracy for arrhythmia detection of a single-lead ECG patch platform featuring self-adhesive dry electrode technology in individuals with chronic coronary syndrome. This sensor was evaluated during exercise and for prolonged, continuous monitoring.

**Methods:**

We recruited a total of 6 consecutive patients with chronic coronary syndrome scheduled for an exercise stress test (EST) as part of routine cardiac follow-up. Traditional 12-lead ECG recording was combined with monitoring with the ECG patch. Following the EST, the participants continuously wore the sensor for 5 days. Intraclass correlation coefficients (ICC) and Wilcoxon signed rank tests were used to assess the utility of detecting arrhythmias with the patch by comparing the evaluations of 2 blinded assessors. Signal quality during EST and prolonged monitoring was evaluated by using a signal quality indicator. Additionally, connection time was calculated for prolonged ECG monitoring. The comfort and usability of the patch were evaluated by a web-based self-assessment questionnaire.

**Results:**

A total of 6 male patients with chronic coronary syndrome (mean age 69.8, SD 6.2 years) completed the study protocol. The patch was worn for a mean of 118.3 (SD 5.6) hours. The level of agreement between the patch and 12-lead ECG was excellent for the detection of premature atrial contractions and premature ventricular contractions during the whole test (ICC=0.998, ICC=1.000). No significant differences in the total number of premature atrial contractions and premature ventricular contractions were detected neither during the entire exercise test (*P*=.79 and *P*=.18, respectively) nor during the exercise and recovery stages separately (*P*=.41, *P*=.66, *P*=.18, and *P*=.66). A total of 1 episode of atrial fibrillation was detected by both methods. Total connection time during recording was between 88% and 100% for all participants. There were no reports of skin irritation, erythema, or pain while wearing the patch.

**Conclusions:**

This proof-of-concept study showed that this innovative ECG patch based on self-adhesive dry electrode technology can potentially be used for arrhythmia detection during vigorous exercise. The results suggest that the wearable patch is also usable for prolonged continuous ECG monitoring in free-living conditions and can therefore be of potential use in cardiac rehabilitation and tele-monitoring for the prevention of exercise-related cardiovascular events. Future efforts will focus on optimizing signal quality over time and conducting a larger-scale validation study focusing on both arrhythmia and ischemia detection.

## Introduction

Higher levels of physical activity and fitness are associated with a lower burden of cardiovascular disease (CVD) [1-4]. However, it is also well established that vigorous exercise is associated with an increased risk of major adverse cardiovascular events in people with underlying CVD. In patients with coronary artery disease (CAD), intense physical activity could lead to fatal ventricular arrhythmias due to plaque rupture or demand ischemia [5,6]. Therefore, the 2019 European Society of Cardiology Guidelines for the diagnosis and management of chronic coronary syndromes state that an exercise electrocardiogram (ECG) provides complementary clinically useful and valuable prognostic information in addition to a resting ECG [7].

In clinical practice, the risk of exercise-induced arrhythmias and ischemia is typically evaluated by a traditional exercise stress test (EST). However, the interpretation of such a test is associated with several limitations. First, an EST usually consists of a single short bout of exercise with a gradually increasing workload (in watts), which is mostly not representative of free-living sports activities in terms of sports type, intensity, and duration. Yet, these factors are particularly important determinants of the occurrence of ischemia and arrhythmias during exercise [5,8,9]. Second, environmental factors such as temperature and hydration status can vary considerably during outdoor sports activities and pose additional risks for patients with CVD [10]. A sensor suitable for continuous ECG monitoring during vigorous exercise can enhance the monitoring of individuals with subclinical or diagnosed CAD in free-living conditions. Such sensors would allow repeated or periodic measurements during exercise, contributing to better screening and management in this group. For individuals engaged in sports, this technology will support health care professionals in providing appropriate and personalized exercise prescriptions.

An extensive review of the literature reveals that there has been a dramatic increase in wearable sensors over the past decade [11-15]. Numerous devices are available for heart rhythm monitoring and arrhythmia detection, most of them based on either ECG or photoplethysmography (PPG) [11]. Generally, for diagnostic purposes, ECG-based wearables are preferred over PPG sensors because ECG-based analyses have been shown to be more accurate than derived analyses based on pulse waveforms. In particular, PPG-based wearables generally lack accuracy for monitoring during exercise [16,17]. Whereas ECG patches are well-tolerated and have high patient adherence [18,19], existing devices such as the Apple Watch, Alivecor Kardia Mobile, or Fibricheck [20-22] often require additional handling to generate an ECG recording, making them unsuitable for continuous use during exercise. These limitations may be overcome by ECG patches, as they maintain direct skin contact, allowing for automatically generated ECG read-outs. Several Conformité Européenne (CE)-marked or Food and Drug Administration–cleared single-use ambulatory ECG patches are currently available [23]. However, research on the usability and accuracy of ECG patches during prolonged (vigorous) sports activities is scarce.

To overcome these barriers, we developed a single-lead wearable vital signs platform featuring self-adhesive dry electrodes with the intended purpose of detecting arrhythmias and myocardial ischemia over prolonged periods of time, including physical activities. The self-adhesive dry electrode technology ensures direct skin contact over many days with minimal skin preparation and no gel application. These features contribute to maintaining good signal quality over time and during exercise, ensuring skin comfort and user compliance. This could make this single-lead wearable patch more suitable for long-term monitoring during physical activity in comparison to gel electrode solutions [24,25]. In this initial proof-of-concept study, we aimed to examine the usability, signal quality, and utility of detecting arrhythmias in ECG signals recorded with the innovative patch during vigorous exercise and for prolonged monitoring in free-living conditions.

## Methods

### Recruitment

#### Study Design and Population

Participants included in this observational proof-of-concept study were adult cardiac patients diagnosed with chronic coronary syndrome who underwent an EST as part of routine follow-up at the Department of Cardiology at Máxima Medical Centre, the Netherlands. Individuals with cardiac pacemakers or other stimulators were excluded, as were participants with an implantable cardioverter defibrillator. Other exclusion criteria were left bundle branch block, Wolff-Parkinson-White syndrome, or ≥0.1 ST-segment depression on resting ECG. All participants performed an exercise test according to standardized exercise testing protocols, in which a traditional 12-lead ECG was recorded. This was combined with monitoring using the ECG patch during the same test. To collect additional data about prolonged ECG recording, the participants were asked to continuously wear the patch for 5 consecutive days. Also, the patients were asked to complete a short web-based questionnaire about the comfort and usability of the patch, which took approximately five minutes to complete.

### Vital Signs Patch Platform

**Figure 1:**
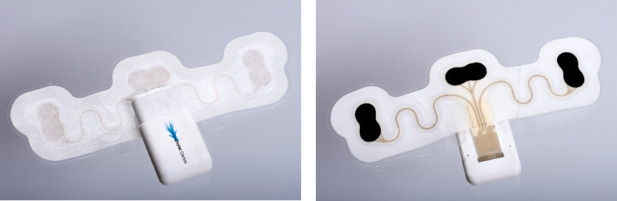
Vital signs patch platform with self-adhesive dry-electrode electrocardiogram (ECG) technology.

In this study, we assessed the utility to detect arrhythmias, signal quality, and usability of the vital signs patch research platform featuring self-adhesive dry electrode technology (Figure 1). The patch platform consists of a disposable patch and a reusable read-out module. The patch contains a pair of electrodes for acquiring a bipolar single-lead ECG signal for continuous monitoring. The printed patch is a layer build-up of conformable thermoplastic polyurethane with flexible and stretchable conductive silver within a meander design for additional strain relief during wear. Self-adhesive and gel-free electrodes are transfer printed onto the design, and a nonwoven acrylic adhesive (MED45150, Avery Dennison) is used as the top layer to ensure dominant skin contact properties for long-term wear durability of the overall patch. The vital signs research platform was developed in the Dutch Organization for Applied Scientific Research (TNO) Holst Centre, and the screen printing was carried out at the Holst Centre manufacturing facilities. The reusable part contains a wireless communication module and read-out electronics for 7 days of continuous monitoring on a single charge (2M Engineering). The patch was placed on the left side of the chest, just below the V4-V6 lead position, shortly before the planned exercise test. The patch records continuously unless the recording is stopped. Sometimes data can be missing, probably due to poor electrode contact. These moments are referred to as disconnection time.

### Study Procedures

#### Exercise Stress Test

The exercise test was performed on a cycle ergometer (Lode Excalibur Sport, Lode BV Medical Technology) as part of routine patient care. According to the local protocol, an individualized ramp protocol was used, aiming for a total test duration of 8-12 minutes. This individualized protocol was based on the predicted maximum workload. The EST consists of an exercise phase with an incremental load and a recovery phase. The patch was applied to the skin of the participant by the investigator. The 12-lead exercise ECG (GE Cardiosoft V6.73, GE Health Care) was monitored continuously throughout the test. Patient characteristics (length and height), maximum workload (Wmax), power-to-weight ratio, and the percentage of predicted maximum heart rate were reported. In patients using beta-blockers, Brawner’s equation was used to predict the maximum heart rate [26].

#### Wearing the Patch in Free-Living Activities

After finishing the EST, the electrodes of the 12-lead ECG were removed, and the participants continued wearing the ECG patch for 5 days. They did not need to make adjustments in their daily activities, but they were not allowed to swim or take a bath. The patch can stay in place during the night and, for instance, while showering. After 5 consecutive days, the patients were instructed to remove the patch themselves. At their scheduled visit to the cardiologist, the participants handed in the device. This visit was scheduled according to standard care, usually within two weeks of the exercise test. In the case of a consultation by phone, the device was picked up at the patient’s home by the investigators. All recorded data was reviewed offline and retrospectively.

### Outcome Measures

#### Arrhythmia Detection

A total of 2 cardiologists, blinded for participant characteristics, assessed all 12-lead and patch ECG recordings in a random order. An evaluation on the following items was performed: the amount of premature atrial contractions (PAC) and premature ventricular contractions (PVC) during the exercise and recovery phases, and the occurrence of supraventricular or ventricular tachycardias. Items for which no consensus was obtained were assessed by a third cardiologist.

#### Signal Quality During Traditional Exercise Testing

Quality analysis was performed using Python (Python Software Foundation). The quality of the ECG recording from the patch during exercise was analyzed using a Signal Quality Indicator (SQI) which resulted in being the best performing one when comparing different SQI metrics with annotated quality levels [27]. This SQI is based on the comparison of successive QRS complexes. To calculate the signal quality, the signal was preprocessed in the same way for all patients with a 0.5 Hz high-pass Butterworth filter. To avoid bias in the analysis, an irregular QRS complexes rejector was included to not affect the estimated quality levels [28]. The output is a number between 0 and 1, with 0 corresponding to the lowest quality and 1 to the highest. Furthermore, the same SQI was applied to the 12-lead ECG signals. The average of the SQIs obtained for each lead was then computed and used for comparison with the SQI results of the patch ECG.

#### Signal Quality for Prolonged Electrocardiogram Recording

We analyzed the quality of the ECG recording from the patch for prolonged monitoring using the connection time and the SQI already mentioned. The connection time of the ECG signal is represented as a percentage. In addition to total time, the time was split into day and night using a regular schedule, from 7 AM to 11 PM and from 11 PM to 7 AM, respectively. The SQI results of the patch ECG during the prolonged monitoring were further processed on all recorded data in order to obtain the percentage of time per day in which the SQI was above 80%. This was then averaged across all participants. Segments of ECG with a quality above 80% are considered high-quality signals [27].

#### Questionnaire

The comfort and usability of the patch were measured using a self-constructed web-based questionnaire in Castor (Castor EDC), a web-based software application for clinical research. The questionnaire consisted of general questions about the wearing time, physical activities while wearing the patch, and an evaluation of whether the patch adhered for the entire period. The following parameters were scored using a 1-5 Likert scale: noticeability of the patch, skin irritation, erythema, and pain (1=totally disagree, 2=disagree, 3=neutral, 4=agree, and 5=totally agree). The removal of the patch was evaluated with 2 questions about the occurrence of pain and skin irritation. An overall comfort score during both the EST and daily activities was asked using a 1-5 Likert scale (1=totally uncomfortable, 2=uncomfortable, 3=neutral, 4=comfortable, and 5=very comfortable).

### Statistical Analyses

Descriptive analyses were conducted for baseline participant characteristics. Continuous data and normal distributed variables are presented as mean (SD). Categorical variables are presented as numbers and percentages. Continuous and nonnormal distributed variables are represented as median (IQR). For the continuous ECG parameters, the degree of agreement between the 2 devices was evaluated using the intraclass correlation coefficient (ICC) with a 95% CI. In this study, an ICC>0.9 was regarded as excellent agreement. To compare the ECG parameters, the Wilcoxon signed rank test was performed. In all statistical analyses, *P*<.05 was considered statistically significant. All statistical analyses were performed using SPSS statistical software (version 22, IBM Corp).

### Ethical Considerations

This study complied with the principles of the Declaration of Helsinki. Ethical approval for this study was waived by the Medical Ethics Review Committee of Máxima Medical Center, Veldhoven, the Netherlands (N22.002), as the rules laid down in the Medical Research Involving Human Subjects Act (also known by its Dutch abbreviation WMO), do not apply to this research. Written informed consent was obtained from all participants when they were enrolled in this study. All data have been deidentified. No compensation was provided to the participants.

## Results

### Participants and Demographics

A total of 6 consecutive patients who fulfilled the inclusion criteria signed the informed consent form and completed the study protocol between May and July 2022. All participants were male, with a mean age of 69.8 (SD 6.2) years. Both patient baseline characteristics and the results of the ESTs are presented in Table 1. All 6 patients completed the EST according to their individualized protocol. All participants were verbally encouraged to exercise until exhaustion, and none of the tests were terminated prematurely. The mean maximum achieved load was 206.1 (SD 96.2) W, and the mean power-to-weight ratio was 2.53 (SD 1.1) W/kg. The mean percentage of the maximum predicted heart rate was 98.8% (SD 14.6%).

**Table 1:** Patient baseline characteristics and exercise test results (n=6).

Characteristic	Values
Age (years), mean (SD)	69.8 (6.2)
Male, n (%)	6 (100)
Height (cm), mean (SD)	178.5 (4.8)
Weight (kg), mean (SD)	80.5 (7.2)
BMI (kg/m^2^), mean (SD)	25.3 (1.8)
Wmax^a^ (Watt), mean (SD)	206.1 (96.2)
Power-to-weight ratio (W/kg), mean (SD)	2.5 (1.1)
%Pred maxHR^b^ (%), mean (SD)	98.8 (14.6)

^a^Wmax: maximum workload.

^b^%Pred maxHR: Percentage Predicted Maximum Heart Rate.

### Arrhythmia Detection

A total of 191 isolated PACs and 296 PVCs were detected in all ESTs using the 12-lead ECG system. The median (IQR) of all detected PACs and PVCs per exercise test is presented in Table 2. The total number of premature complexes during the exercise and recovery phases of the stress test showed an excellent degree of agreement between the 2 ECG recording methods (ICC=0.998, 95% CI 0.982-1.000; ICC=0.998, 95% CI 0.989-1.000; ICC=1.000, 95% CI 0.999-1.000; and ICC=0.998, 95% CI 0.988-1.000). Looking at the total number of PACs and PVCs during the total test duration, the degree of agreement between the 12-lead ECG and the patch ECG is also excellent (ICC=1.000, 95% CI 0.997-1.000, both). There were no significant differences in the total number of PACs and PVCs detected with both methods during the total test duration (*P*=.79 and *P*=.18, respectively), and for the testing stages separately (*P*=.41, *P*=.66, *P*=.18, and *P*=.66).

**Table 2:** Assessment of the number of premature complexes per exercise stress test, expressed as median (IQR). The degree of agreement was evaluated using intraclass correlation coefficients (ICC) with 95% CI.

	12-lead ECG^a^, median (IQR)	Patch ECG, median (IQR)	ICC^b^ (95% CI)	*P* value for mean difference
PAC^c^ exercise	2 (0-18)	1 (0-20)	0.998 (0.982-1.000)	0.41
PAC recovery	0 (0-33)	0 (0-33)	0.998 (0.989-1.000)	0.66
PAC total	2 (0-51)	1 (0-52)	1.000 (0.997-1.000)	0.79
PVC^d^ exercise	2 (0-41)	1 (0-41)	1.000 (0.999-1.000)	0.18
PVC recovery	5 (1-36)	4 (1-36)	0.998 (0.988-1.000)	0.66
PVC total	8 (1-75)	5 (2-74)	1.000 (0.997-1.000)	0.18

^a^ECG: electrocardiogram.

^b^ICC: intraclass correlation coefficient.

^c^PAC: premature atrial contraction.

^d^PVC: premature ventricular contraction.

One patient had an episode of atrial fibrillation during the end of the exercise stage (14 seconds) and during the first part (30 seconds) of the recovery stage. This episode was detected at exactly the same time and for the same duration on both recorded ECGs. No ventricular arrhythmias were detected.

### Signal Quality During Exercise Testing

**Figure 2:**
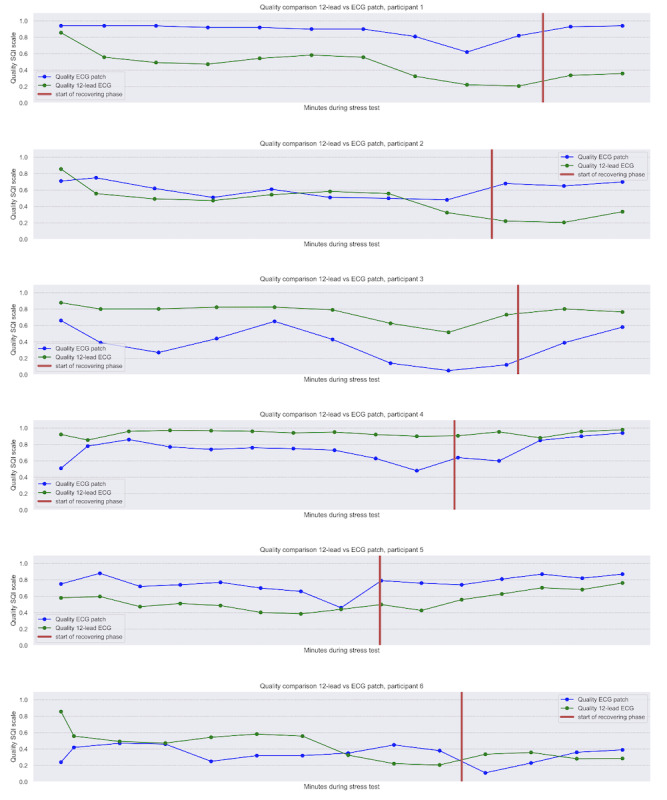
Signal quality index (SQI) of the electrocardiogram (ECG) patch during exercise stress test (EST) based on QRS morphology, compared with the average 12-lead ECG, participants 1-6.

The signal quality of the ECG signal obtained using the patch and 12-lead system during the exercise recordings of all 6 participants is presented in Figure 2. In general, this quality metric shows that the signal quality starts high, decreases to some degree during the EST, and increases again in the recovery phase. In participants 1, 2, and 5, the quality of the ECG patch outperformed the average quality of 12-lead ECG, whereas the opposite was the case in participants 3, 4, and 6. In participants 1, 3, and 5, the relative changes in signal quality followed similar trends for the patch ECG and the 12-lead.

### Signal Quality of the Patch for Prolonged Electrocardiogram Recording

#### Connection Time

Table 3 shows the percentages of connection times in the total recording and those during the day and night. A total of 5 out of 6 participants had a total connection time above 88% (range 88%-100%). The disconnection time during the day was less than 15%, and during the night it was below 4%. In participant 6, the patch was completely disconnected after 52 hours, and no recordings were made after this. In the recording time frame, the connection time of this patch was 100%.

**Table 3:** Percentage of connection time of the patch per person for 5 days of wear.

	Participant 1	Participant 2	Participant 3	Participant 4	Participant 5	Participant 6^a^
Day connection, %	85.65	85.49	87.96	100	99.99	43.72
Night connection, %	97.55	97.56	96.66	100	100	40
Total connection time, %	89.62	88.51	90.86	100	99.90	42.48

^a^In this participant, the patch was completely disconnected prematurely.

#### Signal Quality Indicator

**Figure 3:**
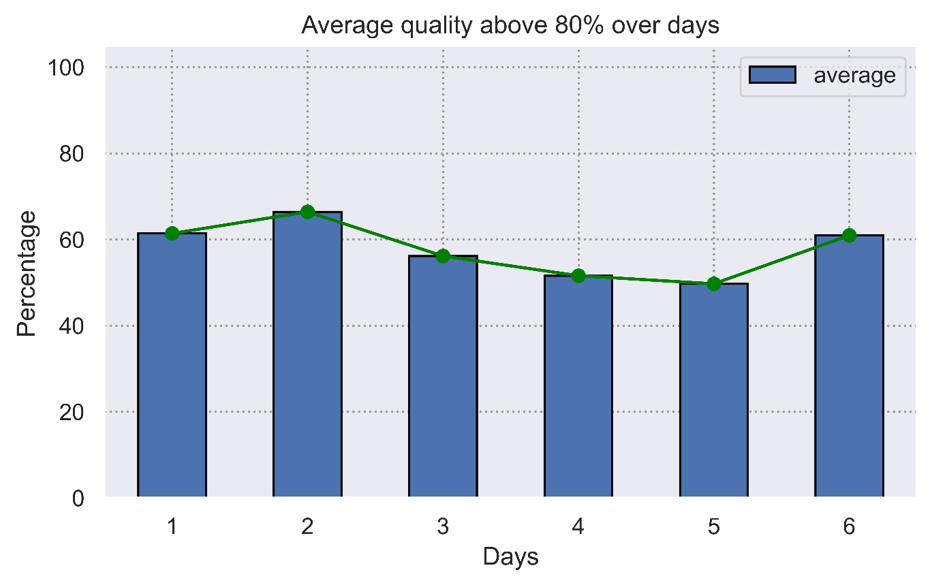
Average percentage of time with high-quality patch electrocardiogram (ECG) signal over the days.

The percentage of time the recorded data has a quality above 80%, average across all participants, is presented in Figure 3, split between days. The average percentage of high-quality signals is similar on days 1 and 6.

### Questionnaire

#### Wearing Time

The average duration of time in which the patch stayed attached to the skin was 118.3 (SD 5.6) hours. There were no issues reported about the loss of adhesion of the patch. Participant 3 removed the patch accidentally on the last day of testing; all other participants wore the patch for at least 5 days (120 hours).

#### User Comfort and Usability

A total of 2 participants found the patch noticeable. There were no reports of skin irritation, erythema, or pain (Figure S1 in Multimedia Appendix 1).

Regarding the removal of the patch, there were also no reports of skin irritation. One participant reported brief pain—less than a minute—when removing the patch. An overall evaluation of the comfort of the patch during the exercise test and in daily life reported no discomfort (Figure S2 in Multimedia Appendix 2).

## Discussion

### Principal Findings

In this proof-of-concept study in 6 cardiac patients, we demonstrated that the vital signs patch platform containing self-adhesive dry electrodes for recording single-lead bipolar ECG can be used for arrhythmia detection during a maximum EST. Additionally, our findings suggest the potential utility of this patch for prolonged, over-5-day ECG monitoring in free-living conditions, although larger-scale validation is necessary. The patch was well tolerated by all participants, and no discomfort or device-associated side effects were reported.

### Interpretation of Findings

Based on our literature review, this is one of the first studies to evaluate the use of an ECG patch with self-adhesive dry electrode technology specifically during exercise, with direct comparison to the traditional 12-lead ECG [15,17]. A patch called the ECG247 Smart Heart Sensor was previously tested in elite endurance athletes during short exercise bouts, but no reference to the gold standard was included [29].

Numerous studies on different ECG patches have been conducted over the past few years. Many of them assessed accuracy for the detection of cardiac arrhythmias at rest or during low-intensity activities, focusing mainly on atrial fibrillation [18,30,31]. Premature atrial and ventricular complexes are often not analyzed. In this study, we assessed the detection of PACs, PVCs, and atrial and ventricular arrhythmias during exercise testing. One participant had an episode of atrial fibrillation during the EST. This episode was assessed by the cardiologists for the same time period and duration on both ECG methods. Although the patch signal quality, based on QRS morphology SQI, decreased during the (sub)maximum load, the patch was still highly accurate when compared with the traditional 12-lead exercise ECG. The signal quality of the ECG patch during exercise therefore appears to be sufficient for rhythm assessment. When comparing the SQI level of the average 12-lead ECG with the ECG patch, the 12-lead ECG performed better in 3 patients during the EST, while the patch outperformed the 12-lead ECG in the other half.

Dry electrode technology offers advantages for prolonged recording compared with gel electrodes, as it causes less skin irritation and erythema [32]. A study that analyzed a dry electrode ECG patch for atrial fibrillation detection reported an overall accuracy of 93.57% and 85.94% during stationary and movement states, respectively [33]. However, the movement state during free-living conditions consisted only of low-intensity activities, such as walking. In this study, the patch stopped recording prematurely in 1 participant. The exact reason this happened is unknown; the patch remained firmly adhered to the skin until its removal on day 5. The wearing time was therefore not affected. This particular failure, as well as the decline in connection times during prolonged wear, may be attributed to issues related to skin-electrode contact or hardware failures. However, the percentage of time with high signal quality in all collected data remained stable over time and was around 60%. Moreover, further analysis of signal quality showed no significant difference in the quality changes between the days [27]. Other research has shown that monitoring for up to 14 days is already feasible with the self-adhesive Zio Patch [18], but the influence of high-intensity activities was not addressed. Prolonged continuous monitoring with a patch device can, however, contribute to higher detection rates in arrhythmia screening compared with conventional 24-hour Holter monitoring [34].

### Limitations

An important limitation of this study was the small number of participants. However, for the purpose of this proof-of-concept study, we considered a sample of 6 patients sufficient to explore the use of the new ECG technology for its feasibility for a larger-scale study and to obtain results to improve the patch and the test procedure. Another limitation is the fact that all patients were male and mainly older adults. The mean BMI of the participants was 25.3 (SD 1.8) kg/m^2^; however, a BMI of <20 kg/m^2^ and a very high BMI of ≥35 kg/m^2^ are risk markers for cardiovascular mortality in patients with chronic coronary syndrome [35]. Future research should expand the participant demographics to include a more diverse group to ensure broader applicability. However, the high user comfort in this group is promising, as older people are more likely to have dry and sensitive skin conditions [36]. Regarding long-term monitoring during physical activities, not all participants in this study were highly active. The adhesion of the patch during extended wear might be different for more active individuals. Furthermore, data for signal quality assessment was missing due to one patch being disconnected prematurely. This, however, did not affect the ability to detect arrhythmias with the patch during exercise. The performance of SQIs based on QRS morphology should be further analyzed for ECG recordings with PACs and PVCs, as the morphology of these extrasystoles is different. Finally, to collect specific information on the comfort and usability of the patch, a self-constructed brief questionnaire was used. For further studies, a more comprehensive questionnaire would provide more information.

### Future Perspectives

Accurate and continuous arrhythmia detection during vigorous exercise could play an important role in primary and secondary cardiovascular prevention in high-risk populations. ECG assessment during physical activities can help monitor individuals with underlying CVD. Arrhythmias during exercise, such as an increasing number of PVCs, can imply exercise-induced myocardial ischemia. Therefore, the patch could be beneficial for monitoring patients with known CAD, but also for screening high-risk athletes and highly active individuals without known CAD, or even patients with underlying structural heart disease who are at risk for fatal arrhythmias.

The ability to detect arrhythmias using this dry electrode ECG technology must be confirmed in a larger-scale validation study with multiple measurements during exercise over prolonged periods of time. There is also added value in exploring patch-based screening for the occurrence of myocardial ischemia during exercise (eg, ST-segment deviation). High signal quality and good skin-sensor contact are essential. Patch design and material selection affect this. Additional preclinical tests with improved patches can contribute to optimizing the patch and reduce the risk of premature disconnection. More research is needed in order to improve the signal quality during prolonged ECG monitoring in a larger heterogeneous cohort and to depict how the signal quality is altered by factors such as gender, body composition, and skin type. This will also add more knowledge about possible causes of connection loss and the potential role of body morphology in this. At the same time, the development of automatic assessment methods is required for clinical application.

As for wearability and comfort, efforts shall include the use of a validated questionnaire on skin irritation and comfort and research on a cohort with wider demographics and body composition (gender, age, hormonal conditions, and BMI), as well as participants with different skin types and highly prevalent skin conditions (sensitive skin and allergic dermatitis).

### Conclusions

This proof-of-concept study using the vital signs patch showed that an ECG sensor based on self-adhesive dry electrode technology has the potential to be useful for arrhythmia detection during vigorous exercise. Our results suggest that the patch is also usable for prolonged ECG monitoring in free-living conditions and can therefore be of potential use in the prevention of exercise-related cardiovascular events. This technology can support health care professionals in telemonitoring solutions and cardiac rehabilitation programs.

## Appendix

Multimedia Appendix 1 This figure shows the distribution of each evaluated item on wearing the patch (n=6). Data are presented as the number of participants who provided a certain answer to each question.

Multimedia Appendix 2 This figure shows the distribution of the overall comfort evaluations (n=6). Data are presented as the number of participants who provided a certain answer to each question.

## Abbreviations

CAD: coronary artery disease
CE: Conformité Européenne
CVD: cardiovascular disease
ECG: electrocardiogram
EST: exercise stress test
ICC: intraclass correlation coefficient
PAC: premature atrial contraction
PPG: photoplethysmography
PVC: premature ventricular contraction
SQI: signal quality indicator
TNO: Dutch Organization for Applied Scientific Research

## Data Availability

The data sets generated during or analyzed during this study are available from the corresponding author on reasonable request.
