# Supplementary figures and images for: The Utility of a Novel Electrocardiogram Patch Using Dry Electrodes Technology for Arrhythmia Detection During Exercise and Prolonged Monitoring: Proof-of-Concept Study
Source: JMIR Form Res. 2023 Nov 30;7:e49346. doi: 10.2196/49346 (PMC10722364; doi:10.2196/49346)

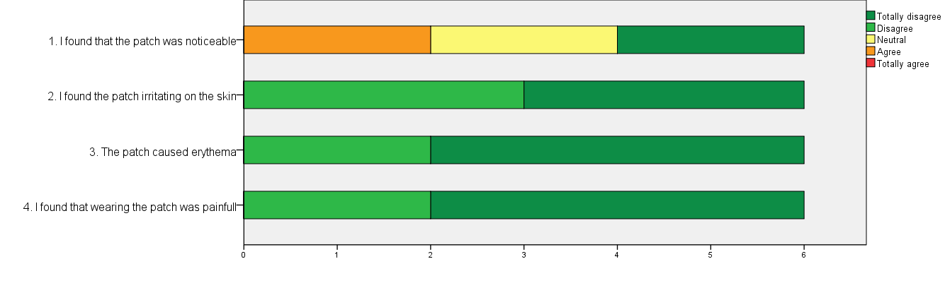

Supplement: Multimedia Appendix 1 [file formative_v7i1e49346_app1.png]

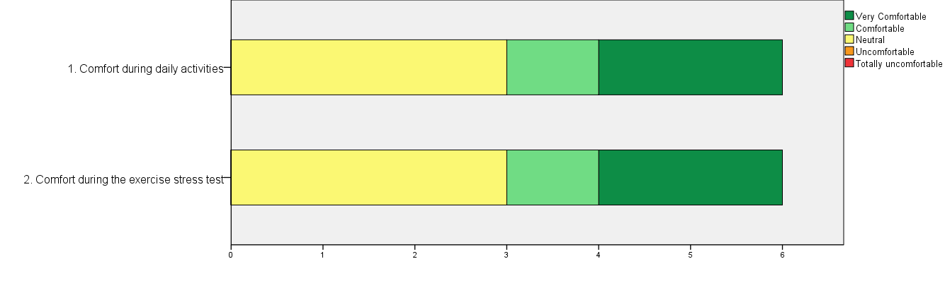

Supplement: Multimedia Appendix 2 [file formative_v7i1e49346_app2.png]
